# Prescriptions for Obesity Medications Among Adolescents Aged 12–17 Years with Obesity — United States, 2018–2023

**DOI:** 10.15585/mmwr.mm7420a1

**Published:** 2025-06-05

**Authors:** Lyudmyla Kompaniyets, Samantha L. Pierce, Renee Porter, Kali Autrey, Kao-Ping Chua, Brook Belay, Heidi M. Blanck, Alyson B. Goodman

**Affiliations:** ^1^Division of Nutrition, Physical Activity and Obesity, National Center for Chronic Disease Prevention and Health Promotion, CDC; ^2^Division of Price Transparency Compliance, Office of Program Operations and Local Engagement, Centers for Medicare & Medicaid Services, San Francisco, California; ^3^School of Public Health, University of Michigan, Ann Arbor, Michigan; ^4^U.S. Public Health Service Commissioned Corps, Rockville, Maryland.

**Summary**

**What is already known about this topic?**

Obesity medications are recommended as part of evidence-based, multicomponent treatment for obesity in adolescents. In 2022, the Food and Drug Administration expanded its approval of two obesity medications to include adolescents aged 12–17 years. In January 2023, the American Academy of Pediatrics released a new clinical practice guideline recommending that clinicians offer obesity medications for adolescents with obesity as an adjunct to health behavior and lifestyle treatment.

**What is added by this report?**

This pharmacoepidemiologic study using ambulatory electronic medical record data found that despite the increasing proportion of adolescents with obesity who were prescribed an obesity medication, <1% were prescribed one in 2023. Prescribing prevalence was higher among girls, White adolescents, those aged 15–17 years, and adolescents with severe obesity.

**What are the implications for public health practice?**

Continued monitoring of the use and safety of obesity medications could guide development and implementation of strategies to ensure that all adolescents have access to evidence-based obesity treatment, including medications and health behavior and lifestyle interventions.

## Abstract

Obesity affects approximately one in five U.S. adolescents. Although an increasing number of medications are approved for adolescent obesity as an adjunct to health behavior and lifestyle treatment, national data on the prevalence and correlates of obesity medication prescribing for adolescents are sparse. Ambulatory electronic medical record data were analyzed to assess trends in the proportion of U.S. adolescents aged 12–17 years with obesity (body mass index ≥95th percentile) who were prescribed Food and Drug Administration (FDA) –approved obesity medications during 2018–2023. Log-binomial models were used to estimate characteristics of adolescents associated with receiving an obesity medication prescription in 2023. The proportion of U.S. adolescents who were prescribed obesity medications increased substantially in 2023 (by approximately 300% compared with 2020), the year after FDA expanded its approval of two obesity medications to include adolescents and after publication of the 2023 American Academy of Pediatrics clinical practice guideline. Despite this substantial relative increase, 0.5% of adolescents with obesity were prescribed an obesity medication in 2023, with a majority (83%) of prescriptions received by adolescents with severe obesity. Semaglutide (Wegovy, indicated for persons aged ≥12 years with obesity), and phentermine or phentermine-topiramate were most commonly prescribed. Prescribing prevalence was higher among girls than among boys (adjusted prevalence ratio [aPR] = 2.05), among adolescents aged 15–17 years than among those aged 12–14 years (aPR = 2.24), and among those with severe (class 2 or class 3) obesity than among those with class 1 obesity (aPR = 4.03 and 12.78, respectively). Prescribing prevalence was lower among Black or African American adolescents than among White adolescents (aPR = 0.61). Continued monitoring of the use of these medications could help guide strategies to ensure that all adolescents with obesity have access to evidence-based obesity treatment, including medications and health behavior and lifestyle interventions.

## Introduction

Approximately one in five U.S. adolescents has obesity.* Obesity is a complex chronic disease that affects a child’s physical, social, and emotional health and increases the risk for adult obesity, type 2 diabetes mellitus (T2DM), and heart disease.^†^ In 2022, the Food and Drug Administration (FDA) expanded its approval of phentermine and topiramate extended-release capsules and semaglutide for chronic weight management in adults to include adolescents aged 12–17 years.^§^ In early 2023, the American Academy of Pediatrics (AAP) released the *Clinical Practice Guideline for the Evaluation and Treatment of Children and Adolescents with Obesity,* which recommended that clinicians offer obesity medications, including glucagon-like peptide-1 receptor agonists (GLP-1RAs), for adolescents aged ≥12 years with obesity as an adjunct to health behavior and lifestyle treatment that facilitates sustained healthier habits, including improved nutrition and physical activity (*1*).

Data on use of obesity medications among adolescents are limited. A 2024 study reported an increase of 504% (among boys) to 588% (among girls) in the number of GLP-1RAs dispensed to U.S. adolescents during 2020–2023 (*2*). The analysis focused on GLP-1RAs, only two of which are FDA approved for obesity treatment in adolescents, and did not ascertain the body mass index (BMI) of participants or assess differences by obesity class (*2*). Another 2024 study found that the 2023 release of the AAP guideline was associated with increases in prescriptions of medications (both FDA-approved obesity medications and those used off-label for obesity treatment) among children and adolescents aged 8–17 years who had obesity but did not have T2DM (*3*). However, the analysis did not describe factors associated with prescribing after release of the guideline. This report, which focuses on FDA-approved obesity medications, assesses trends in prescription prevalence among adolescents with obesity, as well as patient characteristics associated with receiving an obesity medication prescription.

## Methods

### Data Source

IQVIA Ambulatory Electronic Medical Records (EMR) data^¶^ were used to identify U.S. adolescents aged 12–17 years with at least one health care visit during 2018–2023 in which their BMI was recorded as ≥95th percentile for age and sex (i.e., obesity).** Among adolescents included in each year,^††^ this analysis identified those prescribed an FDA-approved obesity medication at least once during the calendar year during which obesity was recorded. Medications included orlistat, phentermine, a combination of phentermine and topiramate (phentermine-topiramate), and setmelanotide, as well as two newly approved high-dose GLP-1RAs: liraglutide (Saxenda, indicated for patients aged ≥12 years with obesity; maximum daily dose = 3 mg, approved in 2020) and semaglutide (Wegovy, indicated for patients aged ≥12 years with obesity; maximum weekly dose = 2.4 mg, approved in 2022). Semaglutide that was indicated only for adults with T2DM (Ozempic; maximum weekly dose = 2 mg) and liraglutide that was indicated for persons aged ≥10 years with T2DM (Victoza; maximum daily dose = 1.8 mg) were not included in the list of obesity medications because they are indicated for T2DM and not for obesity. Semaglutide and liraglutide of undetermined brands were also not included. A sensitivity analysis was performed in which the Ozempic brand of semaglutide, the Victoza brand of liraglutide, and undetermined brands of semaglutide and liraglutide were included.

### 2018–2023 Analysis

The primary study outcome in this multiyear analysis was the presence of a prescription for an obesity medication per patient, per year. Adolescents were considered to have experienced the outcome if they had received an obesity medication prescription at least once during the year that obesity was recorded, and contributed an independent outcome count each year if their prescriptions spanned >1 year. Crude percentages of adolescents prescribed obesity medications were plotted for 2018 through 2023 (total and by medication type). Adjusted percent differences in prescription prevalence in each year (compared with 2020) were obtained from a generalized linear model with log link and binomial distribution to model the outcome variable (any obesity medication prescription) with two possible results (yes or no). The covariate of interest was the year indicator, with the year 2020 selected as a referent for comparison with a published 2024 report that examined the dispensing of GLP-1RAs to adolescents and young adults during 2020–2023 (*2*). The model controlled for age (12–14 and 15–17 years), sex (male and female), and obesity class (classes 1, 2, and 3, with classes 2 and 3 representing severe obesity).^§§^

### 2023 Patient-Level Analysis

Two models were used to conduct the patient-level analysis. Model 1 was a generalized linear model with log link and binomial distribution used to test associations between patient characteristics (i.e., age, sex, obesity class, and U.S. Census Bureau region^¶¶^) and having received at least one obesity medication prescription in 2023. This analysis focused on only 2023 because 1) 2023 represented the period after publication of the AAP clinical practice guideline and the most recent FDA approval of a medication for adolescents with obesity (semaglutide [Wegovy]), 2) 2023 was the most recent complete year with available obesity medication data in IQVIA, and 3) obesity medication prescription rates before 2023 were very low. A secondary analysis using the same model tested the association between covariates (age, sex, and U.S. Census Bureau region) and presence of severe obesity (class 2 or 3). Estimates of association from the model were expressed as adjusted prevalence ratios.

Models did not adjust for race and ethnicity because these data were missing for 26.1% of the sample. Model 2 was restricted to the subpopulation of patients who were Black or African American (Black) or White and included an additional covariate of race (Black/White). Analyses were conducted using R software (version 4.4.1; The R Foundation) and Stata (version 15.1/MP; StataCorp). This activity was reviewed by CDC, deemed research not involving human subjects, and was conducted consistent with applicable federal law and CDC policy.***

## Results

### Prevalence of Adolescents Who Received Obesity Medication Prescriptions

**FIGURE 1:**
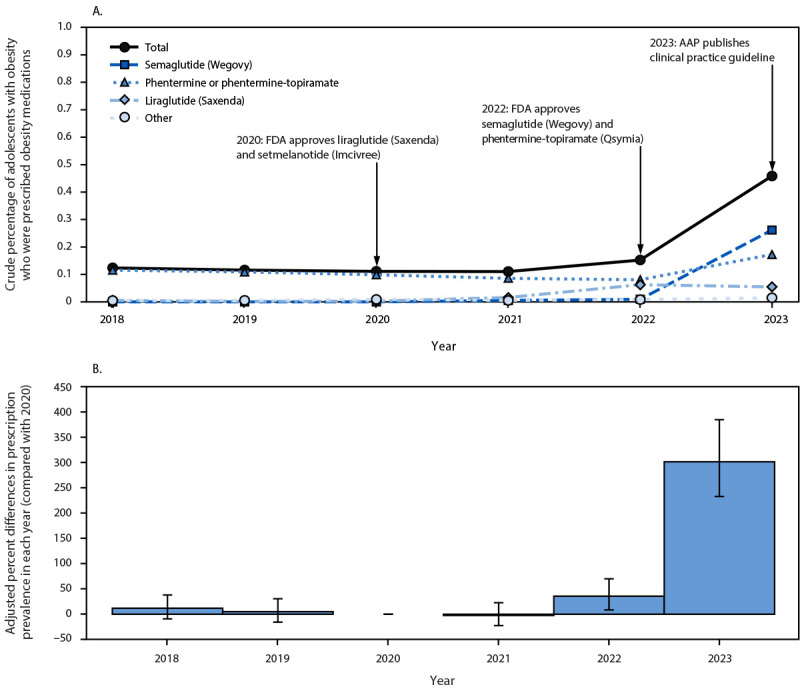
Crude percentages (A) and adjusted percent differences in prevalence compared with 2020 (B)* of adolescents aged 12–17 years with obesity who received an obesity medication prescription^†^ — IQVIA Ambulatory Electronic Medical Records, United States, 2018–2023^§^ **Abbreviations:** AAP = American Academy of Pediatrics; BMI = body mass index; FDA = Food and Drug Administration. * Adjusted percent differences in prescription prevalence in each year (compared with 2020) were obtained from a generalized linear model with log link and binomial distribution. The adjusted model controls for sex, age category, and obesity class. Obesity was defined as BMI ≥95th percentile for age and sex. 95% CIs indicated by bars. ^†^ In November 2020, FDA approved setmelanotide (Imcivree) for treating obesity in persons with monogenic or syndromic obesity aged ≥6 years. In December 2020, FDA approved liraglutide (Saxenda) for treating obesity in adolescents aged ≥12 years. In June 2022, FDA approved phentermine-topiramate (Qsymia) for treating obesity in adolescents aged ≥12 years. In December 2022, FDA approved semaglutide (Wegovy) for treating obesity in adolescents aged ≥12 years. In January 2023, a new AAP clinical practice guideline recommended that clinicians offer obesity medications as part of evidence-based multicomponent treatment for adolescents aged 12–17 years with obesity (AAP Clinical Practice Guideline for the Evaluation and Treatment of Children and Adolescents With Obesity). ^§^ The sample included 526,973 U.S. adolescents aged 12–17 years with obesity who had a total of 789,057 annual BMI measurements during 2018–2023.

Among 526,973 U.S. adolescents aged 12–17 years with obesity (789,057 person-years) during 2018–2023, the crude percentage of adolescents with obesity who received obesity medication prescriptions was low overall, increasing from 0.1% in 2020 to 0.2% in 2022, then increasing sharply to 0.5% in 2023 (Figure 1). Compared with 2020, the adjusted proportion of adolescents with obesity who received an obesity medication prescription was 301.7% higher (95% CI = 232.8%–385.0%) in 2023. In 2023, 57.1% of adolescents with obesity who were prescribed obesity medications received a prescription for semaglutide (Wegovy), followed by phentermine or phentermine-topiramate (37.7%), liraglutide (Saxenda) (11.9%), and others (3.3%) (Table). A sensitivity analysis that included semaglutide and liraglutide (regardless of indication) showed a slightly higher total prevalence (0.7% in 2023) (Supplementary Figure 1).

**TABLE:** Percentage of adolescents aged 12–17 years with obesity* who were prescribed obesity medications, by selected demographic characteristics, obesity class, and medication type — IQVIA Ambulatory Electronic Medical Records, United States, 2023

Characteristic	No. of adolescents with obesity (%)	No. of adolescents prescribed obesity medication (%)
**Total**	**93,121 (100)**	**427 (100)**
**Age group, yrs, mean (SD)**	**14.4 (1.7)**	**15.2 (1.6)**
12–14	49,584 (53.2)	139 (32.6)
15–17	43,537 (46.8)	288 (67.4)
**Sex**		
Female	41,683 (44.8)	266 (62.3)
Male	51,438 (55.2)	161 (37.7)
**Race**		
Asian	1,203 (1.3)	5 (1.2)
Black or African American	12,192 (13.1)	47 (11.0)
White	51,900 (55.7)	254 (59.5)
Other	3,506 (3.8)	14 (3.3)
Unknown	24,320 (26.1)	107 (25.1)
**U.S. Census Bureau region** ^†^		
Northeast	9,372 (10.1)	23(5.4)
South	22,994 (24.7)	132 (30.9)
Midwest	46,243 (49.7)	186 (43.6)
West	14,497 (15.6)	86 (20.1)
Unknown	15 (—)	0 (—)
**Obesity class** ^§^		
Class 1	55,073 (59.1)	73 (17.1)
Class 2 (severe obesity)	25,019 (26.9)	133 (31.1)
Class 3 (severe obesity)	13,029 (14.0)	221 (51.8)
**Obesity medication^¶^**		
None	—	0 (—)
Semaglutide (Wegovy)	—	244 (57.1)
Phentermine or phentermine-topiramate	—	161 (37.7)
Liraglutide (Saxenda)	—	51 (11.9)
Other (orlistat or setmelanotide)	—	14 (3.3)

**Abbreviation:** BMI = body mass index.

* Obesity was defined as BMI ≥95th percentile for age and sex.

^†^ *Northeast*: Connecticut, Maine, Massachusetts, New Hampshire, New Jersey, New York, Pennsylvania, Rhode Island, and Vermont; *Midwest*: Illinois, Indiana, Iowa, Kansas, Michigan, Minnesota, Missouri, Nebraska, North Dakota, Ohio, South Dakota, and Wisconsin; *South*: Alabama, Arkansas, Delaware, District of Columbia, Florida, Georgia, Kentucky, Louisiana, Maryland, Mississippi, North Carolina, Oklahoma, South Carolina, Tennessee, Texas, Virginia, and West Virginia; and *West*: Alaska, Arizona, California, Colorado, Hawaii, Idaho, Montana, Nevada, New Mexico, Oregon, Utah, Washington, and Wyoming.

^§^ Obesity classes: class 1 (BMI ≥95th percentile to <120% of the 95th percentile), class 2 (BMI of 120% to <140% of the 95th percentile), and class 3 (BMI ≥140% of the 95th percentile).

^¶^ More than one obesity medication could have been prescribed.

### Characteristics of Adolescents Who Received Obesity Medication Prescriptions

In 2023, among 93,121 adolescents with obesity, a total of 38,048 (40.9%) had severe obesity, including 25,019 (26.9%) and 13,029 (14.0%) with class 2 and class 3 obesity, respectively. Among 427 adolescents with at least one obesity medication prescription in 2023, a total of 354 (82.9%) had severe obesity, including 133 (31.1%) and 221 (51.8%) with class 2 and class 3 obesity, respectively (Table).

**FIGURE 2:**
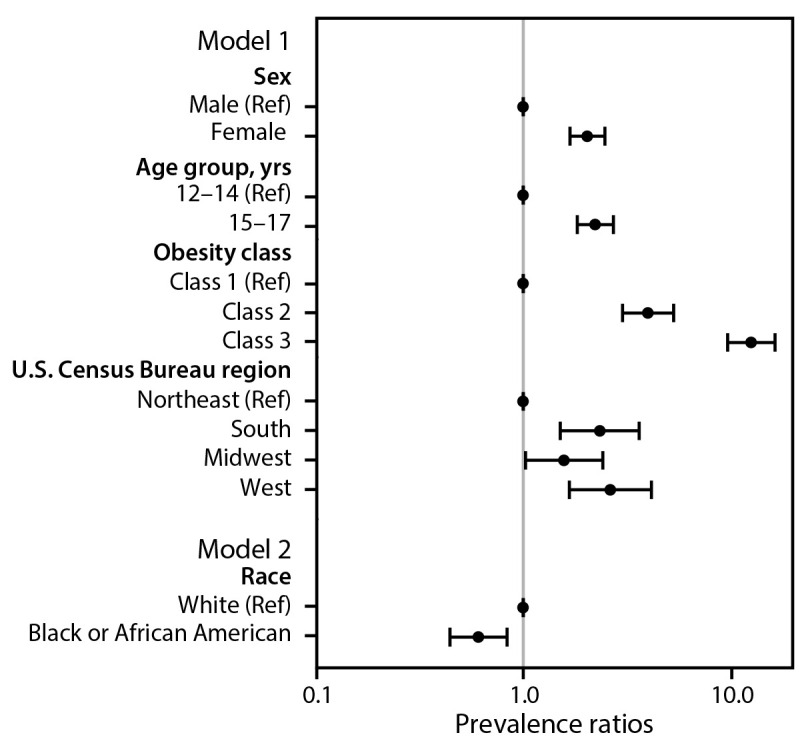
Adjusted prevalence ratios* for receiving an obesity medication prescription among adolescents aged 12–17 years with obesity,^†^ by selected demographic characteristics and obesity class^§^ — IQVIA Ambulatory Electronic Medical Records, United States, 2023 **Abbreviations:** BMI = body mass index; Ref = referent. * 95% CIs indicated by bars. ^†^ Obesity was defined as BMI ≥95th percentile for age and sex. ^§^ A generalized linear model with log link and binomial distribution (model 1) was used to estimate characteristics associated with the outcome of receiving an obesity medication prescription in 2023: age (12–14 years [Ref] and 15–17 years), sex (male [Ref], female), obesity class (class 1 [Ref], class 2, and class 3), and U.S. Census Bureau region (Northeast [Ref], South, Midwest, and West). Model 2 was restricted to adolescents who were Black or African American (Black) or White and included the same covariates as model 1, with an additional covariate of race (Black/White). Obesity classes were as follows: class 1 obesity or BMI ≥95th percentile to BMI <120% of the 95th percentile [Ref], class 2 obesity or BMI of 120% to <140% of the 95th percentile, and class 3 obesity or BMI ≥140% of the 95th percentile. Classes 2 and 3 represented severe obesity. Estimates of association from the model were expressed as adjusted prevalence ratios and plotted on a log(10) scale.

In adjusted analyses, compared with referent populations (boys, adolescents aged 12–14 years, and residents of the Northeast region), adolescents were more likely to be prescribed obesity medications if they were girls (adjusted prevalence ratio [aPR]  =  2.05; 95% CI  = 1.69–2.49), aged 15–17 years (aPR = 2.24; 95% CI  = 1.83–2.74) and lived in the West region (aPR = 2.65; 95% CI = 1.68–4.19), South region (aPR = 2.35; 95% CI  = 1.51–3.65), or Midwest region (aPR = 1.58; 95% CI  = 1.03–2.43) (Figure 2). Adolescents with class 2 obesity were more likely than those with class 1 to be prescribed obesity medications (aPR = 4.03; 95% CI = 3.03–5.36), as were those with class 3 obesity (aPR = 12.78; 95% CI = 9.82–16.64). 

One half (50.0%) of Black adolescents with obesity in 2023 had severe obesity, compared with 39.0% of White adolescents (Supplementary Table). When the analysis was restricted to 12,192 Black adolescents and 51,900 White adolescents, Black adolescents were 39% less likely to be prescribed obesity medications than White adolescents (aPR = 0.61; 95% CI = 0.44–0.84) (Figure 2), despite being 27% more likely to have severe obesity (aPR = 1.27; 95% CI = 1.24–1.29) (Supplementary Figure 2). 

## Discussion

This pharmacoepidemiologic study using a large ambulatory EMR database detected a substantial relative increase (approximately 300%) in the proportion of U.S. adolescents with obesity who were prescribed an obesity medication in 2023, which was the year after FDA expanded its approval of two obesity medications to include adolescents^†††^ and after publication of the AAP clinical practice guideline in January 2023 (*1*). Despite this substantial relative increase, <1% of adolescents with obesity were prescribed an obesity medication in 2023, with a majority (83%) of prescriptions received by adolescents with severe obesity.

A recent study reporting prescriptions filled from 93.6% of U.S. retail pharmacies showed an approximate 500%–590% increase in the dispensing of GLP-1RAs to adolescents aged 12–17 years between 2020 and 2023 (*2*). This report, which focuses on prescribing of FDA-approved obesity medications, including 2 GLP-1RAs, for adolescents, demonstrated a lower but still substantial (approximately 300%) increase, indicating rising use of multiple classes of obesity medications. A recent study also reported an increase in prescribing of FDA-approved and off-label medications among children and adolescents with obesity after publication of the AAP clinical practice guideline in 2023 (*3*). Given this increase in prescriptions, postmarketing monitoring is essential to track potential increases in unanticipated side effects or adverse events associated with the use of these medications (*4*). Because of recent GLP-1RA shortages, safety concerns also might arise for persons filling prescriptions with counterfeit medications or compounded medications (formulations that are created for specific patients or settings, rather than for commercial distribution, and that are not FDA approved); the safety, effectiveness, and quality of these products are not evaluated by FDA before dispensation to the patient (*5*).^§§§^ All adolescents with obesity, including those who receive obesity medications, should receive evidence-based health behavior and lifestyle interventions, which can help them and their families build skills that promote healthier nutrition, physical activity, and related behaviors; lower their health risk; and improve quality of life and self-esteem (*1*).^¶¶¶^ This study could not elicit data on whether adolescents were also receiving these recommended interventions. Public health and health care organizations might need to assess their capacity and readiness to provide these evidence-based interventions to the millions of U.S. children and families who need them.

Semaglutide indicated for persons aged ≥12 years with obesity (Wegovy) and phentermine or phentermine-topiramate were the most prescribed obesity medications in 2023. The oral administration, lower out-of-pocket costs, and more consistent availability of phentermine or phentermine-topiramate (compared with semaglutide, which is administered by weekly subcutaneous injections) might be factors in the increased use among adolescents in 2023 compared with previous years (*6*).

This study excluded medications that were not FDA approved for obesity treatment in adolescents but are often used off-label for this purpose (e.g., metformin, semaglutide [Ozempic], and liraglutide [Victoza], all indicated for persons with T2DM). A sensitivity analysis including semaglutide and liraglutide regardless of indication resulted in a slightly higher prescription prevalence (0.7% in 2023). Future analyses could focus on medications prescribed off-label for obesity or for other conditions that also help with weight management.

The findings in this report indicate that health care providers tended to prescribe obesity medications to adolescents with severe obesity. Approximately 83% of adolescents who received an obesity medication prescription had severe obesity (class 2 or 3), including 52% with class 3 obesity. Higher obesity class is associated with increased cardiometabolic risk, lower health-related quality of life, and declines in physical function (*7*,*8*), which might prompt providers to prescribe obesity medications to this population.

Prescribing of obesity medications also differed by sex, race, and U.S. Census Bureau region. Girls were more likely than boys to be prescribed obesity medications. In addition, although the prevalence of severe obesity among Black adolescents was 27% higher than among White adolescents, Black adolescents were 39% less likely than White adolescents to receive an obesity medication prescription. Factors that might explain differences in prescribing or low prescription rates include limited availability of the medications because of production shortages (*9*), high out-of-pocket costs (*6*), and insurance restrictions, such as lack of coverage or complex prior authorization processes.**** In addition, concerns among adolescents and health care providers about long-term use and safety, as well as health care provider knowledge and self-efficacy in prescribing obesity medications, could impact prescription rates (*10*).

### Limitations

The findings in this report are subject to at least five limitations. First, although this analysis included a geographically diverse sample of health care–seeking adolescents with measured height and weight, the sample was not representative of all U.S. adolescents; this analysis should be replicated with other datasets, particularly those that are population based. Second, although prescriptions documented in ambulatory EMR data were able to be tracked, some prescriptions might have been provided in outpatient visits that were not captured in this database. Third, although prescribing behaviors were tracked, information about whether the medications were dispensed or used was not available. Fourth, missing data on race and ethnicity limited the ability to examine differences in obesity medication prescribing. Finally, this analysis did not adjust for household income, insurance status, or other factors that might be associated with receiving an obesity medication prescription.

### Implications for Public Health Practice

Despite the increasing proportion of adolescents with obesity who were prescribed an obesity medication from 2018 to 2023, <1% were prescribed one in 2023. Continued monitoring of the use and safety of these medications in adolescents, as well as barriers to availability and access, could help guide the development and implementation of strategies to ensure that all adolescents have access to evidence-based obesity treatment, including medications and health behavior and lifestyle interventions.

## Footnotes

* Obesity is defined as body mass index (kg/m^2^) ≥95th percentile for sex and age.

†
CDC. About Obesity

§
Food and Drug Administration. New Drug Therapy Approvals 2022

¶ Observational Medical Outcomes Partnership (OMOP), version 5, May 2024 data release. IQVIA’s ambulatory EMR database includes structured clinical data for approximately 90 million health care–seeking patients, including 15 million children, from all 50 states. Data come from a single electronic health record vendor that collects data from approximately 100,000 health care providers who are affiliated with approximately 800 medium to large ambulatory practices and physician networks across the United States. All data were extracted using IQVIA’s Health Data Engine, a web-based software as a service platform (Real World & Health Data Sets - IQVIA).

** Measured height and weight data during 2018–2023 for adolescents aged 12–17 years in IQVIA were cleaned longitudinally using growthcleanr, an open-source R package (version 2.2.0; R Core Team) for cleaning pediatric growth data (GitHub - carriedaymont/growthcleanr). Height and weight measurements were merged using a 30-day nearest-neighbor rolling join and cleaned cross-sectionally using CDC growth charts (exclusions: height-for-age z-score of <−5 or >5; weight-for-age z-score <−10 or >5; BMI-for-age z-score of <−4 or >10; and BMI >150 kg/m^2^).

†† The highest BMI per calendar year per person was used to calculate sex-specific BMI-for-age percentiles for 2018–2023. The final sample included adolescents with obesity (BMI ≥95th percentile). Pregnant adolescents were excluded. The study identified 526,973 adolescents with obesity who had a total of 789,057 annual BMI measurements.

§§ Obesity classes are as follows: class 1 obesity or BMI ≥95th percentile to BMI <120% of the 95th percentile [referent group], class 2 obesity or BMI of 120% to <140% of the 95th percentile, and class 3 obesity or BMI ≥140% of the 95th percentile. Classes 2 and 3 represent severe obesity.

¶¶ *Northeast*: Connecticut, Maine, Massachusetts, New Hampshire, New Jersey, New York, Pennsylvania, Rhode Island, and Vermont; *Midwest*: Illinois, Indiana, Iowa, Kansas, Michigan, Minnesota, Missouri, Nebraska, North Dakota, Ohio, South Dakota, and Wisconsin; *South*: Alabama, Arkansas, Delaware, District of Columbia, Florida, Georgia, Kentucky, Louisiana, Maryland, Mississippi, North Carolina, Oklahoma, South Carolina, Tennessee, Texas, Virginia, and West Virginia; and *West*: Alaska, Arizona, California, Colorado, Hawaii, Idaho, Montana, Nevada, New Mexico, Oregon, Utah, Washington, and Wyoming.

*** 45 C.F.R. part 46; 21 C.F.R. part 56; 42 U.S.C. Sect. 241(d), 5 U.S.C. Sect. 552a, 44 U.S.C. Sect. 3501 et seq.

††† Food and Drug Administration. Wegovy Highlights of Prescribing Information; Food and Drug Administration. QSYMIA (phentermine and topiramate extended-release capsules for oral use) Highlights of Prescribing Information

§§§ Food and Drug Administration. FDA warns consumers not to use counterfeit Ozempic (semaglutide) found in U.S. drug supply chain; Food and Drug Administration. Human Drug Compounding Laws

¶¶¶
CDC. CDC-Recognized Family Healthy Weight Programs

****
Institute for Clinical and Economic Review. Affordable Access to GLP-1 Obesity Medications: Strategies to Guide Market Action and Policy Solutions
